# Graft failure after allogeneic hematopoietic stem cell transplantation in pediatric patients with acute leukemia: autologous reconstitution or second transplant?

**DOI:** 10.1186/s13287-024-03726-z

**Published:** 2024-04-22

**Authors:** Tahereh Rostami, Mohammad Reza Rostami, Amir Hossein Mirhosseini, Saeed Mohammadi, Mohsen Nikbakht, Hediyeh Alemi, Naghmeh Khavandgar, Soroush Rad, Ghasem Janbabai, Seied Asadollah Mousavi, Azadeh Kiumarsi, Amir Kasaeian

**Affiliations:** 1 Hematologic Malignancies Research Center, Research Institute for Oncology, Hematology and Cell Therapy, Shariati Hospital, Tehran University of Medical Sciences, Tehran, Iran; 2 Department of Internal Medicine, School of Medicine, Imam Ali Hospital, Alborz University of Medical Sciences, Alborz, Iran; 3 Cell Therapy and Hematopoietic Stem Cell Transplantation Research Center, Research Institute for Oncology, Hematology and Cell Therapy, Shariati Hospital, Tehran University of Medical Sciences, Tehran, Iran; 4 Hematology, Oncology and Stem Cell Transplantation Research Center, Research Institute for Oncology, Hematology and Cell Therapy, Shariati Hospital, Tehran University of Medical Sciences, Tehran, Iran; 5 Digestive Oncology Research Center, Digestive Diseases Research Institute, Shariati Hospital, Tehran University of Medical Sciences, Tehran, Iran; 6 Department of Pediatrics, School of Medicine, Childrens Medical Center, Tehran University of Medical Sciences, Tehran, Iran; 7 Clinical Research Development Unit, Shariati Hospital, Tehran University of Medical Sciences, Tehran, Iran

**Keywords:** Graft failure, Myeloablative conditioning, Hematopoietic stem cell transplantation, Salvage transplantation, Acute leukemia, Disease-free survival

## Abstract

**Background:**

Graft failure (GF) is a rare but serious complication after allogeneic hematopoietic stem cell transplantation (HSCT). Prevention of graft failure remains the most advisable approach as there is no clear recommendation for the best strategies for reversing this complication. Administration of growth factor, additional hematopoietic progenitor boost, or a salvage HSCT are current modalities recommended for the treatment of GF. Autologous recovery without evidence of disease relapse occurs rarely in patients with GF, and in the absence of autologous recovery, further salvage transplantation following a second conditioning regimen is a potential treatment option that offers the best chances of long-term disease-free survival. The preconditioning regimens of second HSCT have a significant impact on engraftment and outcome, however, currently there is no consensus on optimal conditioning regimen for second HSCT in patients who have developed GF. Furthermore, a second transplant from a different donor or the same donor is still a matter of debate.

**Observations:**

We present our experience in managing pediatric patients with acute leukemia who encountered graft failure following stem cell transplantation.

**Conclusions and relevance:**

Although a second transplantation is almost the only salvage method, we illustrate that some pediatric patients with acute leukemia who experience graft failure after an allogeneic stem cell transplant using Myeloablative conditioning (MAC) regimen may achieve long-term disease-free survival through autologous hematopoiesis recovery.

## Introduction

The successful outcome of hematopoietic stem cell transplantation (HSCT) in the treatment of malignant and non-malignant diseases relies on stable donor hematopoietic cell engraftment, which restores functional hematopoiesis and achieves immunological reconstitution. However, failure to establish persistent engraftment after HSCT remains a significant factor contributing to morbidity and mortality. Graft failure (GF) is a rare but significant complication following allogeneic HSCT, with varying incidences depending on the type of donor [1–3].

Graft failure is classically divided into primary and secondary failure. Primary graft failure is defined as the absence of initial donor cell engraftment by day + 28 if the graft source is peripheral blood (PB) or bone marrow (BM), and by day + 42 if the graft source is umblical cord blood (UCB). Secondary graft failure is characterized by the loss of donor cells after initial engraftment. Table 1 presents the definitions of hematopoietic recovery, graft rejection and failure, poor graft function, and donor chimerism in allogeneic stem cell transplantation [4–6]**.**

**Table 1:** Definitions of hematopoietic recovery, graft rejection, graft failure, poor graft function, and donor chimerism in allogeneic hematopoietic cell transplantation [7–10]

Term	Definition	
Graft failure^*^	Lack of hematopoietic cell engraftment after allogeneic or autologous HSCT	
Primary graft failure (PrGF)	*MAC allo-HSCT*	*Graft source is peripheral blood (PB) or bone marrow (BM)*: Failure to achieve a threshold absolute neutrophil count (ANC) of 0.5 × 10^9^/L for 3 consecutive days by day 28 after HSCT with associated pancytopenia and absence of initial donor cell engraftment (donor cells less than 95%; Mixed or full recipient chimerism)
		*Graft source is umbilical cord blood (UCB):* Failure to achieve a threshold absolute neutrophil count (ANC) of 0.5 × 10^9^/L for 3 consecutive days by day 42 after HSCT with associated pancytopenia and absence of initial donor cell engraftment (donor cells less than 95%; Mixed or full recipient chimerism)
	*RIC allo-HSCT*	ANC < 0.5 × 10^9^/L by day + 28/ + 42, and assay confirming ≥ 5% donor type cells Failure to surpass the 5% donor type threshold, even if essentially normal blood counts
Secondary graft failure	Loss of previously functioning graft (may involve hemoglobin and/or platelets and/or neutrophils) associated with loss of full donor chimerism RIC allo-HSCT: Loss of donor hematopoiesis to < 5%	
Graft rejection	GF caused by an immune-mediated process; rejection of donor cells mediated by host cells	
Poor graft function (PGF)	Severe cytopenia of at least two cell lines and/or frequent dependence on blood and/or platelet transfusions and/or growth factor support with full donor chimerism Absence of other explanations such as disease relapse, drugs or infections *Poor graft function* Primary PGF: incomplete hematological recovery Secondary PGF: decrease of blood counts after prompt recovery	

^*^An isolated cytopenia does not necessarily invoke GF, as this may represent a transitory phenomenon related to a medication, viral infection, a lineage‐specific immune‐mediated cytopenia, or graft‐versus‐host disease (GvHD). MAC Myeloablative conditioning; RIC Reduced intensity conditioning

**Fig. 1:**
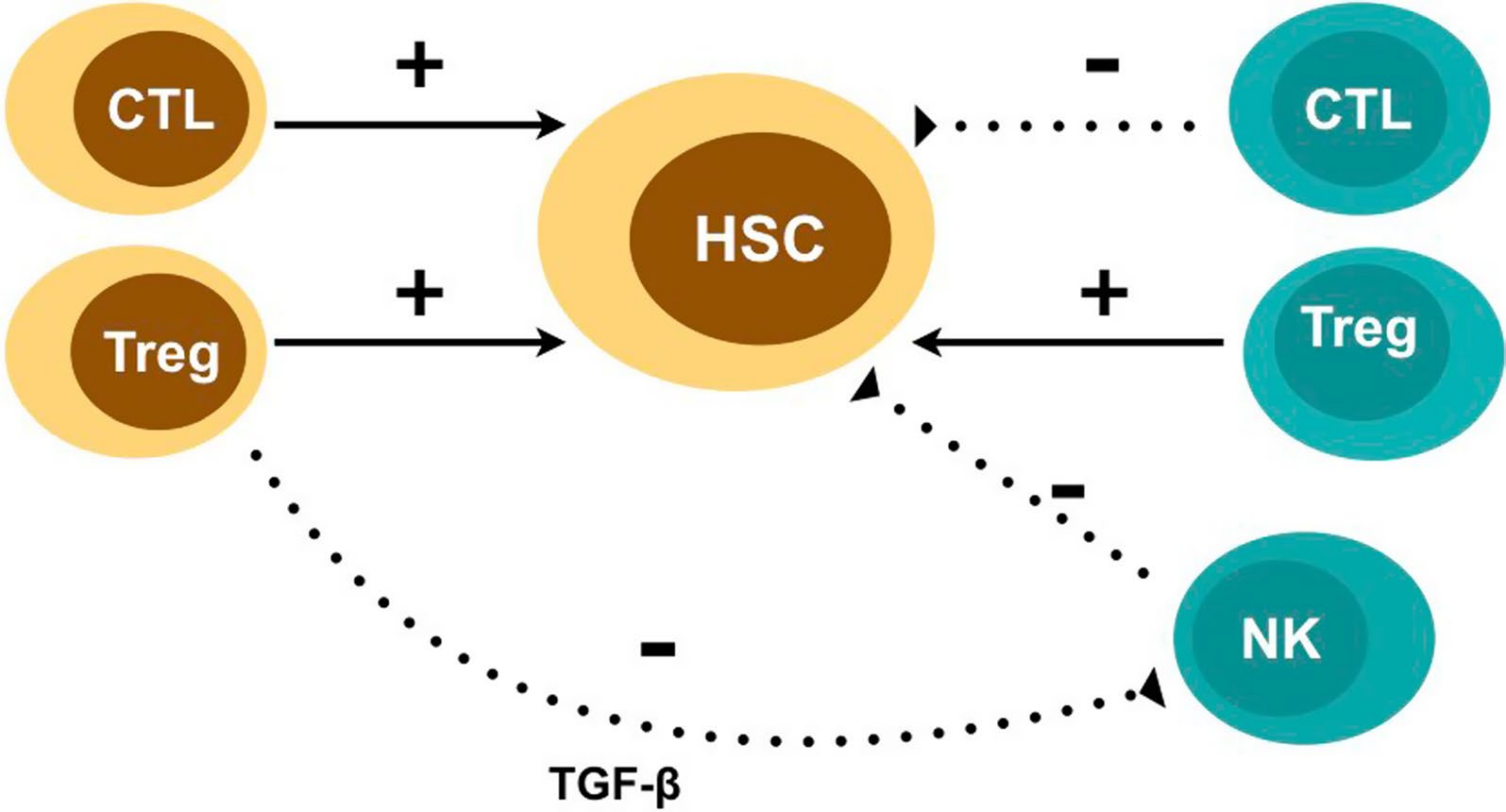
Immunological basis of graft failure

GF is relatively uncommon in patients with leukemia who undergo HSCT from a Human leukocyte antigens (HLA) matched related donor. However, it is more commonly observed in patients with non-malignant diseases and those who receive alternative donor stem cell transplants, with incidences ranging from 4% in HLA matched unrelated donors to 20% in transplants from UCB [4, 11–15]. It is more frequent following haploidentical-HSCT, with an incidence of around 10% in T cell-depleted grafts, 13% in the era of post-transplant cyclophosphamide (PTCY), and 1% in Beijing protocol [16]. Promotion and failure of engraftment occure as an interaction between recipient and donor cytotoxic T lymphocytes (CTL), regulatory T cells (Tregs; CD4 + CD25 + Foxp3 + regulatory T cells) and Natural killer (NK) cells. Graft failure occurs as the result of a classical alloreactive immune response driven by residual host immunity persisting following a preparative regimen. The most prominent effector cells that induce GF are thought to be residual host CTL [7]. Conversely, donor cytotoxic T cells promote HSC engraftment. Therefore, a T-cell deficient graft would be associated with a higher prevalence of GF (Fig. 1) [17–19].

Recipient or donor Tregs are crucial immunomodulatory cells that provide interactions between immune and hematopoietic cells, and both are important in facilitating engraftment. Donor Tregs promote engraftment by mediating NK cell suppression, and Host Tregs help hematopoietic stem cells to maintain in the bone marrow niche [7, 20, 21]. Recipient Tregs ablation by anti-CD25 monoclonal antibodies (mAbs) has been strongly associated with inhibition of allogeneic rejection, and accordingly, adoptive transfer of host-type Tregs enhances engraftment [21–24]. NK cells represent an important part of the innate immune system and alloreactive NK cells promote engraftment following HLA-haploidentical HSCT. However, residual recipient NK cells can eliminate donor hematopoietic stem cells through perforin-mediated cytotoxicity, and result in graft rejection [7, 25, 26].

Although preventing graft failure is the most advisable approach, [3] there is no clear consensus on the best strategies to reverse this complication. However, some potential approaches to manage graft failure include the administration of growth factors, waiting for autologous reconstitution (AR), providing an additional hematopoietic progenitor boost, or undergoing a second transplant with a second preparative regimen [27]. Several reports have suggested that a second salvage transplant for graft failure in children can lead to significant transplant-related mortality and seriously compromise overall survival due to prolonged periods of aplasia when the recipient is at a higher risk of infection and hemorrhage [27]. Different factors may affect the outcome of second transplant in pediatric patients.

This review outlines our approach to this complication using our illustrative pediatric patients with acute leukemia who experienced primary and secondary GF. Additionally, it discusses the risk factors for graft failure, various approaches to manage it, salvage transplant procedure including selection of the best conditioning regimens and most appropriate donor, and also waiting for autologous recovery.

### Patient 1: T cell acute lymphoblastic leukemia (T Cell ALL) with primary GF (Second transplant from a different donor)

**Fig. 2:**
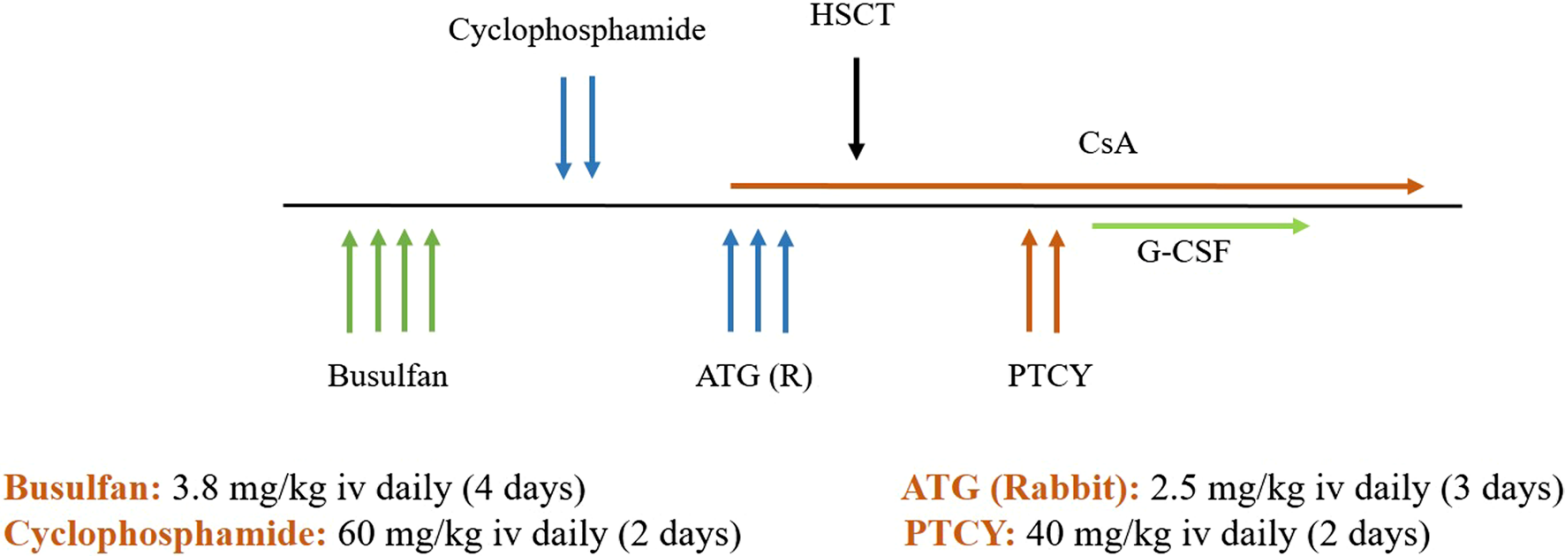
Conditioning regimen for haploidentical hematopoietic stem cell transplant

A 9-year-old boy diagnosed with T Cell ALL received HLA-haploidentical donor stem cell transplantation in his second complete remission, from his 40-year-old father with T-cell replete granulocyte colony-stimulating factor (G-CSF) mobilized peripheral blood stem cells (PBSC) as the graft source and a cell dose of 6.3 × 10^6^/kg CD34 + cells. The preparative regimen consisted of busulfan (16 doses; 3.8 mg/kg/day) and cyclophosphamide (a total dose of 120 mg/kg). Rabbit anti-human thymocyte globulins (ATG-Thymoglobuline, 2.5 mg/kg/day, from days − 3 to − 1) were added before transplant to prevent rejection. The graft versus host disease (GvHD) prevention included PTCY (a total dose of 80 mg/kg) plus cyclosporine A (Fig. 2). The patient and donor were ABO match and both CMV seropositive. Pre-transplantation donor-specific antibodies (DSA) were negative. On day + 28 post-HSCT, the patient's white blood cell (WBC) and platelet count were 0.1 × 10^9^/L and 15 × 10^9^/L, respectively, with hypoplastic bone marrow in morphologic remission. Donor chimerism by short tandem repeat polymerase chain reaction (STR-PCR) technique was also less than 5%.

**Fig. 3:**
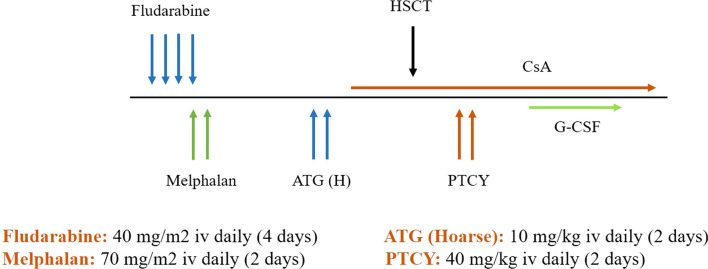
Conditioning regimen for second transplant

Once primary graft failure was diagnosed, cyclosporine A was withdrawn, DSA was reassessed, and salvage HSCT was planned immediately. Performance status according to the Lansky Play-Performance scale was 90%. However, the selection of the optimal donor source and a safe conditioning regimen was of utmost importance due to the occurrence of BK virus-induced hemorrhagic cystitis. He underwent a second transplant 37 days after diagnosis of GF from his haploidentical mother (different donor) with T-cell replete G-CSF mobilized PB hematopoietic stem cells as graft source and a cell dose of 10 × 10^6^/kg CD34 + cells. An immunoablative RIC regimen consisting of fludarabine (40 mg/m2/day × 4 days), melphalan (70 mg/m2/day × 2 days), and ATG (Hoarse; 10 mg/kg/day × 2 days) was used. PTCY (40 mg/kg/day × 2 days) and cyclosporine A administered to prevent acute and chronic GvHD (Fig. 3). Neutrophil and platelet engrafted on days + 11 and + 14 respectively with complete donor chimerism on days + 28 post-salvage transplant. BK viremia and viruria cleared post-engraftment. On the last follow-up, 18 months after re-transplant, bone marrow is in complete morphologic remission, donor chimerism by STR-PCR is 100% and measurable residual disease (MRD) level by multiparametric flow cytometry is negative. However, the patient developed steroid-resistant skin chronic GvHD and responded well to ruxolitinib.

### Patient 2: acute myeloid leukemia (AML) with secondary GF (Rescue transplant from different donor)

A 3-year-old boy with high-risk AML in first complete remission received HLA-haploidentical donor allogeneic stem cell transplant from his 37-year-old father with T-cell replete G-CSF mobilized PBSC as graft source and a cell dose of 9 × 10^6^/kg CD34 + cells. The conditioning and GvHD prophylaxis were similar to patient 1 (Fig. 2). The patient and donor were ABO match and both CMV seropositive. Engraftment occurred successfully with a complete donor chimerism. On day + 25 he presented with fever. WBC Count was 0.3 × 10^9^/L accompanied by hypoplastic marrow and loss of full donor chimerism (less than 5%). Blood culture was positive for pseudomonas aeruginosa and DSA was negative. With the diagnosis of secondary graft failure, immunosuppressive drugs tapered off and broad-spectrum antibiotics started. Re-transplant was planned from another parent (his mother) with a RIC regimen (Fig. 3) with a cell dose of 10 × 10^6^/kg CD34 + cells/kg. He achieved neutrophil and platelet engraftment with complete donor chimerism on day + 28 of post-salvage transplant. He experienced steroid-resistant skin chronic GvHD that was resolved by ruxolitinib. On the last follow-up, 3 years after the rescue transplant, BM is in complete morphologic remission with full donor chimerism.

### Patient 3: precursor B cell acute lymphoblastic leukemia (pre B Cell ALL) with secondary GF (rescue transplant from the same donor)

A 7-year-old boy with Pre B Cell ALL in the second remission received HLA-haploidentical donor HSCT from his 13-year-old sister with T-cell replete G-CSF mobilized PBSC as graft source and a cell dose of 4.5 × 10^6^/kg CD34 + cells. The conditioning regimen and GvHD prophylaxis were similar to patient 1 (Fig. 2). Myeloid and platelet engraftment occurred on days + 12 and + 19, respectively. Donor chimerism by STR-PCR was 100% on day + 28. On day + 40, he was hospitalized due to fever, vomiting, and malaise. WBC Count was 0.2 × 10^9^/L accompanied by hypoplastic marrow and loss of complete donor chimerism (< 5%). CMV reactivation was detected by plasma sample using real-time PCR with a viral load of more than 2 × 10^6^ million-copy number/ml, and blood culture was positive for pseudomonas aeruginosa, and DSA was negative. As soon as secondary graft failure was diagnosed, immunosuppressive drugs tapered off, broad-spectrum antibiotics and antifungals started, and for CMV reactivation, foscarnet was prescribed. On day + 60 post-transplant, the patient was afebrile with a negative blood culture test, and CMV by real-time PCR was also undetectable. However, the WBC count was still 0.2 × 10^9^/L, and donor chimerism by STR-PCR still reported less than 5%. Second HSCT was planned from the same donor with the RIC regimen as described in patient 1 (Fig. 3) with a cell dose of 8 × 10^6^/kg CD34 + cells/kg. Neutrophil and platelet engraftment occurred on days + 13 and + 17, respectively, with complete donor chimerism on days + 28 post-salvage transplant. Unfortunately, one year after transplant, he experienced bone marrow relapse.

### Patient 4: philadelphia chromosome-positive (Ph +) precursor B cell acute lymphoblastic leukemia (pre B Cell ALL) with primary GF (autologous hematopoiesis recovery)

A 6-year-old boy diagnosed with Ph + Pre B Cell ALL in second remission received HLA-matched unrelated donor allogeneic HSCT with bone marrow hematopoietic stem cells as graft source and a cell dose of 3 × 10^6^/kg CD34 + cells. The preparative regimen consisted of busulfan (16 doses; 3.8 mg/kg/day) and cyclophosphamide (a total dose of 120 mg/kg). Rabbit anti-human thymocytes globulins (ATG-Thymoglobuline, 2.5 mg/kg/day, from days − 3 to − 1) were added before transplant to prevent rejection. The GvHD prophylaxis included cyclosporine A and a short course of methotrexate. Pre-transplantation MRD level by Real Time Polymerase chain reaction (RT-PCR) for BCR-ABL1/ABL1 was undetectable, and DSA was negative. On day + 28 post-HSCT, WBC and platelet count were 0.1 × 10^9^/L, and 10 × 10^9^/L respectively, with hypoplastic bone marrow and donor chimerism by STR-PCR of less than 5%. Once the diagnosis of primary graft failure was established, cyclosporine A tapered off and as additional stem cells were unavailable, a conservative approach including growth factor was adopted while awaiting hematological recovery. He developed autologous hematological reconstitution 57 days after the transplant. Prophylactic Imatinib started, and MRD was assessed by RT-PCR (BCR-ABL1/ABL1) every three months.

Three years after the transplant, he feels well, and his bone marrow is in complete morphologic and molecular remission with a donor chimerism by STR-PCR of less than 5%.

### Patient 5: precursor B cell acute lymphoblastic leukemia (pre B Cell ALL) with primary GF (autologous hematopoiesis recovery)

An 8-year-old girl diagnosed with Pre B Cell ALL with BM relapse underwent HLA-haploidentical donor HSCT from his 35-year-old father with T-cell replete G-CSF mobilized with a cell dose of 9 × 10^6^/kg CD34 + cells.

Pre-transplantation DSA was negative. The patient and donor were ABO minor mismatch and both CMV seropositive. CMV reactivation was detected by plasma sample using real-time PCR on day + 4 and cleared by foscarnet on day + 17. On day + 28 post-transplant, WBC and platelet count were 0.1 × 10^9^/L and 10 × 10^9^/L respectively, with hypoplastic bone marrow and absence of donor chimerism.

Once the diagnosis of primary graft failure was confirmed, cyclosporine A was tapered off. Our preference in the same situations is a rescue second transplant with a RIC as soon as possible. However, her parents refused to accept a second transplant, and a conservative approach was adopted. She developed autologous hematological reconstitution 45 days after the transplant. MRD was assessed by multiparameter flow cytometry every three months and on the last follow-up, two years after GF, she is well, in complete morphologic remission with undetectable MRD, with and donor chimerism by STR-PCR of less than 5%.

## Discussion

With the increasing number of patients eligible for allogeneic HSCT, only 25–30% of patients have an HLA-identical sibling donor, and finding a suitable HLA-compatible unrelated volunteer donor is possible for less than 70% of the remaining patients [28, 29]. In the absence of an HLA-matched donor, alternative donors of hematopoietic stem cells (HSCs), such as unrelated UCB and HLA-haploidentical relatives, are being increasingly used [30, 31]. This means that more patients may experience graft failure.

Between August 2015 to june 2022, five pediatric patients with acute leukemia (4 with ALL and 1 with AML) experienced graft failure (3 primary and 2 secondary) after allogeneic hematopoietic stem cell transplantation (HSCT) in our center. All patients had received haploidentical stem cell transplants, except for one who had been transplanted from a matched unrelated donor (MUD). Table 4 displays the characteristics of our patients who developed graft failure. Recently donor-specific antibodies (DSA) against nonshared, either major or minor donor histocompatibility antigens have been found to predict primary GF (2- to tenfold increase) in HLA haploidentical mismatched family transplants, especially in multiply transfused patients [3]. HLA disparity between the donor and recipient in haploidentical transplantation can contribute to bidirectional alloreactivity, both in graft-versus-host and host-versus-graft directions, which increases the risk of developing Primary GF [32, 33].

Although PTCY overcomes T cell- and NK cell-mediated graft rejection, antibody-mediated rejection by DSAs appears to be one of the principal mechanisms of primary GF [33]. DSAs target donor HLA antigens present on the surface of hematopoietic progenitor cells. Consequently, antigen–antibody complexes bind to C1q and activate the complement cascade, resulting in the formation of a membrane attack complex that causes donor cells lysis that leads to allograft rejection [34].

### Risk factors of graft failure

Conditions associated with increased occurrence of graft failure include defects in the bone marrow microenvironment, immunological disturbances or imbalances between donor and recipient (HLA disparity, alloimmunization with anti-HLA antibodies, ABO mismatching in the donor/recipient pairs, etc.), low infused hematopoietic stem cell dose, T-cell depleted (TCD) grafts, reduced-intensity conditioning regimens (RIC), drug toxicity (myelosuppressives such as ganciclovir) and infections, especially of viral origin, such as those caused by cytomegalovirus (CMV) [16, 35–37].

The risk of Primary GF after haploidentical HSCT has been reported from 1% with myeloablative conditioning to 8% with non-myeloablative preparative regimens, from both BM or PB as stem cell source [38]. Moreover, in hematological malignancies, GF occurs more frequently in patients with a high-risk disease due to intensive or prolonged chemo/radiotherapy before transplant because of damage to the bone marrow microenvironment [3]. Pre-transplant transfusion-induced alloimmunization may also affect donor engraftment [39]. Table 2 illustrates conditions associated with an increased risk of graft failure.

**Table 2:** Risk factors for graft failure [3, 8, 40–42]

Immunologic risk factors	Disease/Patient/Donor related	Graft characteristics
HLA disparity between donor and recipient (Haploidentical > MUD & MMD > MSD)	Underlying disease (Non-malignant; Aplastic anemia, Hemoglobinopathies > Malignant)	Graft source (cord blood > bone marrow > mobilized pripheral blood)
Presence of pre-HSCT donor specific antibodies (DSAs)	Advanced disease in hematologic malignanicies	Low CD34 + cell dose
Graft manipulation (Ex-vivo T Cell depletion)	Extensive marrow fibrosis; Myelofibrosis	Storage techniques (cryopreservation)
Intensity of conditioning regimen (RIC > MAC)	Splenomegally (MPD, MDS)	
Major ABO incompatibility	Extensive pre‐transplantation chemotherapy and/or irradiation	
History of extensive transfusion	Iron overload	
Infections (Viral)	Advanced recipient age	
Graft versus Host Disease (GvHD)	Advanced donor age	
Post‐transplantation immune suppression regimen	Female donor grafts for male recipients	

MAC Myeloablative conditioning; MDS myelodysplastic syndrome; MMD Mismatched donor; MPD Myeloproliferative disease; MSD Matched sibling donor; MUD Matched unrelated donor; RIC Reduced intensity conditioning

### Donor-specific anti-HLA antibodies

Donor-directed anti-human leukocyte antigen (HLA)- specific alloantibodies (DSAs) are preformed IgG antibodies against the unshared HLA molecules with the donor [43]. The strong association between DSA and graft failure after mismatched unrelated donors, cord blood, and haploidentical transplantation has been demonstrated [32, 34, 44–46]. Patients may form DSA as a consequence of exposure to foreign cells or a tissue, including pregnancy, previous blood product transfusion, and history of organ or blood transplantation [40]. Although DSAs against HLA class I (HLA-A and HLA-B) and class II (HLA-DRB1) antigens have an unfavorable effect on engraftment, the role of anti-HLA Abs against HLADPB1 and HLA-DQB1 is still unclear [41].

Due to high HLA disparities, the prevalence of DSA in recipients of haploidentical HSCT is higher than in matched unrelated donors, mismatched unrelated donors (mMUD), and UCB transplants [33]. Females with multiple pregnancies have a higher mean fluorescent intensity (MFI) value of DSA (86%), compared to male recipients (5%), as a consequence of alloimmunization after pregnancies against offspring antigens [47]. Several studies have shown that higher MFI values of DSA that represent the “strength” of the antibodies have been associated with an increased rate of graft failure. Although there is not a clear cut-off consensus above which the DSA is likely to cause graft failure [48, 49], in a study by Nordlander A et al., 75% of patients with MFI > 1500 before haploidentical HSCT experienced GF compared to 5% of patients without DSA [50]. Hence, frequent monitoring of DSA levels is necessary, as it is used to determine the need for pre-or post-transplant desensitization and as a decision point to consider an alternative donor against whom the patient has no DSAs, including other haploidentical related donors, UCB, and or 9/10 matched unrelated donor [40, 47, 49]. If post-transplant GF is due to DSA, second transplantation from the same donor would be at risk of engraftment failure. Therefore for retransplant in this setting, a different donor should be considered [32].

### Treatment of graft failure

The management of graft failure can vary depending on center preference and experience. If autologous recovery is not possible, salvage HSCT following a second conditioning regimen is often considered the best option for achieving long-term disease-free survival (DFS) [51]. This approach aims to shorten the period of bone marrow aplasia and reduce the associated risks of infection and hemorrhage. The outcome of salvage HSCT is dependent on the comorbidities that the patient has experienced from the first HSCT [52].

The graft failure rate after salvage transplant is still high, and stable engraftment has been reported as low as 33% in the literature [51, 53]. Nevertheless, the survival rate of patients with GF after allogeneic HSCT without a second salvage transplantation is dismal, at only < 10% [54].

The preconditioning regimens of salvage transplant have a significant impact on engraftment and outcome [50]. However, there is currently no consensus on the optimal conditioning regimen for a second HSCT in patients who have developed GF. Most transplant centers prefer a non-myeloablative regimen that maintains sufficient immunosuppressive effects to eradicate residual host cells to promote engraftment and lessen excessive toxicity, given that patients are very fragile early after the first transplantation. On the other hand, myeloablative conditioning seems unnecessary as bone marrow is already hypocellular [8, 55–57].

Although different donor sources have been used for rescue HSCT after GF [58]**,** transplantation from an immediately available donor is the optimal therapeutic option. Shortening the delay in donor procurement is of particular importance.

Several centers prefer G-CSF-mobilized PBSCs to bone marrow-derived stem cells as graft sources for salvage HSCT due to their higher engraftment rate. PBSCs have advantages such as a larger number of stem cells and higher T-cell content, which can lead to improve graft-versus-tumor effects. However, PBSC transplantation is also associated with an increased risk of GvHD [53, 59].

UCB is another important stem cell source for immediate HSCT, as it is readily available [50]. Waki et al. [60] evaluated 80 adult patients who received UCB transplants within 3 months of GF. In multivariate analysis, conditioning with fludarabine plus alkylating agents and the infusion of cord blood containing ≥ 2.5 × 10^7^ /kg cells were associated with a higher probability of engraftment. However, transplantation-related mortality on day 100 was 45%, with 60% related to infectious complications, demonstrating the need for the earlier application of cord blood before patients complicated by infection or organ toxicity.

### Second HSCT

There is no clear recommendation for the best approach to primary or secondary graft failure. No single drug or strategy has been proven to be superior to others for reversing graft failure, and current approaches to limit the detrimental impact of this complication are primarily based on its prevention. The rescue strategies are limited, and the most common approaches include recombinant growth factors (if it has not been already started as a scheduled treatment protocol), reinfusion of autologous frozen backup progenitors (if available, depending on center policy), waiting for autologous hematopoiesis recovery and salvage HSCT [61].

Survival after a second transplant has been reported to be between 10 and 30% in retrospective studies, mostly due to the poor performance of patients with GF [62]. Several key factors may contribute to successful second transplantation including a safe conditioning regimen, a short interval between GF and re-transplantation, selection of the optimal donor source, and also patient's performance status. Patients with uncontrolled active infection or GvHD, significant organ dysfunction, and poor performance are excluded from salvage transplant [32, 63].

Regarding the optimal conditioning regimen for salvage transplant, several reports have shown favorable outcomes following fludarabine-based reduced-intensity conditioning regimens [64]. A short-term reduced-intensity conditioning regimen, known as a 'one-day regimen’ including alemtuzumab developed to enhance immunosuppression by T-cell depletion. However, alemtuzumab has been associated with an increased risk of infections. Excluding alemtuzumab and a combination of fludarabine and low–dose total body irradiation (TBI) defined as a 'modified one-day conditioning regimen’ has been successful in achieving stable neutrophil engraftment [57, 65].

Fludarabine in combination with ATG as a non-myeloablative regimen and a higher number of CD34 + hematopoietic stem cells has been also associated with consistent hematopoietic reconstitution in patients with GF [51]. The immunoablative reconditioning regimens with fludarabine-based protocols and the use of a different haploidentical donor have been represented as a realistic option to rescue pediatric patients with GF [66]. Furthermore, the incorporation of alkylating agents in a preparative regimen for the second transplant has been related to survival [50]. So, a combination of alkylating agents with fludarabine may contribute to a better outcome.

We have also found that using fludarabine and melphalan as a RIC preparative regimen for a second transplant is appropriate when retransplantation is considered soon after the occurrence of graft failure.

Most of these patients lack a well-matched related or unrelated donor readily available and searching for unrelated volunteer donors from the registry bank is not practical due to the urgent need for preparation of the donor [7, 61]. In recent years, haploidentical HSCT outcomes have improved due to advances in HLA typing, GvHD prophylaxis with PTCY, wide availability of multiple donors, and also supportive care. Haploidentical progenitors are considered a valid alternative for patients who lack a suitable source of progenitors (same donor, backup progenitors, another compatible donor) for a second transplant [66, 67]. On the other hand, some studies have shown that longer intervals between graft failure and rescue transplant can be associated with better survival, probably due to enough time for recovery from first transplant-related toxic complications [67]. Guardiola et al. [68] reported that an inter-transplant time interval of more than 80 days (relative risk: 0.38, 95% confidence interval: 0.19 ± 0.76, P = 0.01) was associated with significantly improved outcomes in patients with primary or secondary graft failure.

Three of our patients underwent salvage hematopoietic stem cell transplantation after an interval from the first HSCT of 37, 52, and 78 days. None of them experienced significant transplant-related complications, and in all of them, hematological recovery occurred successfully. Table 3 displays several selected studies using the second transplant in patients with graft failure.

**Table 3:** Selected studies using second transplant for patients with graft failure

Author	NO	Underlying disease	Donor	Conditioning regimen	Median time between first and salvage HSCT	GvHD prophylaxis	Stem cell source	CI of Neutrophil engraftment	Overall survival (OS
Nagler et al. [69]	243	Malignant	Same donor in 49% and different in 51%	RIC in 80.4% and MAC in 19.6%	48 days (28–120)	PTCY: 27.7% In vivo T-cell depletion: 49.1% Ex vivo T-cell depletion: 13.5%	-	73.7%	5-year: 30.7%
Suma et al. [64]	10	Malignant	UCB	FLU /CY /TBI	38.5 days (35–46)	Tacrolimus /MMF	UCB	80%	1-year: 50.0%
Sun et al. [32]	13	Malignant	Different HID	FLU/CY	49 days (35–120)	Basixilimab/cyclosporine A/MMF	PBSC/BMSC	100%	1-year: 56.6%
Subburaj et al. [70]	4	Malignant/ non-malignant	HID/ same donor (1 patient) or another haplo family donor (3 patients)	FLU/CY	54 days (45–65)	Tacrolimus /MMF	PBSC	100%	With a median follow up time of 28.5 months (range 1 –69 months): 50%
Sabrina Giammarco et al. [71]	20	Malignant	HID/ same donor (13 patients) or another haplo family donor (6 patients)	Baltimore protocol	42 days (34–82)	PTCY,/Cyclosporine/MMF	PBSC	74%	1-year: 66%
Harada et al. [72]	699	Malignant/ non-malignant	HID/UCB	MAC (N = 16) RIC (N = 528) NMA (N = 160)	HID: 42 days (17–757) UCB: 42 days (19–2250)	CNI + MTX/MMF/ prednisolone/ ATG	UCB	HID: 79.7% UCB: 52.5%	1-year OS, 33.1 vs. 34.6% for the HID and UCB groups
Kongtim et al. [53]	31	Malignant	HID/ 7/19 (36.8%) from the same HID	FLU/CY/TBI	48 days (27–147)	PTCY,/Tacrolimus /MMF	-	87.5%	FLU-CY-TBI regimen: 42.9% Other regimens: 6.7% (P = 0.043)
Pedro Prata et al. [67]	24	Malignant/ non-malignant	HID/ same donor (N = 4) Different HID (N = 20)	FLU-based RIC	63 days (39 to 98)	PTCY/Cyclosporine ± MMF	PBSC/BMSC	79%	1-year: 56% (95% CI, 38% to 81%)
Mochizuki et al. [73]	6	Malignant	Same donor (N = 1) Different donor (N = 5)	RIC: FLU/ATG FLU/MEL/ATG	37.5 days (28–126)	Tacrolimus/MTX/Prednisolone	BMSC (N = 5) PBSC (N = 1)	All but one patient	4 are alive and in sustained remission for more than 4 years at the time of the last follow-up
Christelle Ferrà [27]	49	Malignant/ non-malignant	Same donor (N = 38) Different donor (N = 37) autologous back-up (N = 5)	FLU /ATG CY/ATG ATG	69 days (24–652)	Cyclosporine, Cyclosporine/MMF Cyclosporine/Prednisone Cyclosporine/MTX Tacrolimus/MTX CD34 + selection No prophylaxis	PBSC/BMSC/UCB	80% (95% CI: 69–91)	5-year probability of survival: 31% (95%CI: 18%-44%)
Singh et al. [61]	12	Malignant	HID/UCB	FLU /CY/TBI FLU/BU FLU /CY/Alemtuzumab	41 days (31–64)	Tacrolimus/Sirolimius Cyclosporine /MMF Tacrolimus/MTX/ATG PTCY /Tacrolimus/ MMF	UCB/BMSC	9/12	3-year OS: 37%
Kato et al. [50]	102	Malignant/ non-malignant	MMD/UCB	FLU/CY MEL/ATG /Irradiation	39.5 days (18–59)	-	-	55.7 ± 5.0%	1-year: 53.3 ± 5.0%
Fuji et al. [54]	220	Malignant/ non-malignant	1-locus mismatched donor, HID, Matched donor	None (N = 13) MAC (N = 5) RIC (N = 122) NMA (N = 77)	Time from graft failure to transplantation:11 (0–89) days	-	CB (N = 180) BMSC (N = 16) PBSC (N = 24)	UCB: 39% PBSCs: 71% BMSC: 75%	1-year OS was 58% with PBSCs, 38% with BMSC, and 28% with UCB
Lang et al. [66]	11	Malignant/ non-malignant	HID/ different donor	TLI (TBI) /FLU/ATG and/or OKT3 (N = 10) One patient received CY instead of irradiation, TT was added in 6 patients	40 days (31–106)	CD3/CD19 depleted grafts; Patients, whose grafts contained more than 2.5 × 10^4^ T cells/kg bodyweight received pharmacological GvHD prophylaxis with MMF	PBSC	100%	8/11 patients are disease free (median follow up 1.9 years; 1 year-EFS = 72%)
Chewning et al. [51]	16	Malignant/non-malignant	Same donor (N = 6) Different donor (N = 10) Related donor (N = 11) Unrelated donor (N = 5) Matched donor (N = 5) Mismatched donor (N = 11)	FLU (N = 1) FLU/CY (N = 9) FLU/TT (N = 5)	45 days (31–85)	eATG (N = 8) rATG (N = 3) Alemtuzumab (Campath) (N = 3 p) TCD (N = 2) Cyclosporine A ± MMF or steroids (N = 6) MTX (N = 1) Steroids alone (N = 1)	BMSC (N = 3) PBSC (N = 13)	100%	3-year: 35%

ATG Anti thymocyte globuline; BMSC Bone marrow stem cell; CNI Calcineurin inhibitor; CY Cyclophosphamide; eATG Equine ATG; FLU Fludarabine; GvHD Graft versus Host Disease; HID Haploidentical donor; MAC Myeloablative conditioning; MEL Melphalan; MMD Mismatched donor; MMF Mycophenolate mofetil; MTX Methotrexate; NMA Non myeloablative; OKT3 Muromonab-CD3; PBSC Peripheral blood stem cell; PTCY Post-transplantation cyclophosphamide; rATG Rabbit ATG; RIC Reduced intensity conditioning; TBI Total body irradiation; TCD T-Cell depleted; TLI Total lymphatic irradiation; TT Thiotepa; UCB Umblical cord blood

Giammarco et al. [71] reported 19 patients with primary graft failure after haploidentical HSCT who received a second transplant. There was no statistically significant difference in the hematopoietic reconstitution rate between the patients who received a graft from the same donor (77%) and patients transplanted from another haploidentical family donor (66%) (P = 0.5). In an observational study using data from the Center for International Blood and Marrow Transplant Research (CIBMTR) database on unrelated donor transplants, 122 patients with graft failure underwent a second transplant of whom 98 patients grafted from the same donor and 24 from a different donor. One-year overall survival after the second transplant was dismal (11%), and the long-term outcome was not different between patients who transplanted from the same or different donors [74].

In a series by Grandage et al. [75] 12 pediatric patients (< 18 years) who received ex vivo T cell-depleted marrow from unrelated donors suffered graft failure (five primary, seven secondary), of whom seven patients received a second transplant from a different unrelated donor. However, the source did not affect the outcome of the second HSCT (Table 4).

**Table 4:** Summary of our patients characteristics

Patient NO	Type of GF	Source of stem cell in first HSCT	Approach	The interval between first and second transplant	Donor for re-transplant	Time to recovery	Disease status on follow-up	Survival status
1	Primary	PBSC	Re-transpllant	37 days	Different	–	Remission	Alive
2	Secondary	PBSC	Re-transpllant	52 days	Different	–	Remission	Alive
3	Secondary	PBSC	Re-transpllant	78 days	Same	–	Relapse	Dead
4	Primary	BMSC	AR	–	–	57 days	Remission	Alive
5	Primary	PBSC	AR	–	–	45 days	Remission	Alive

AR: Autologous Reconstitution; BMSC: Bone Marrow Stem Cell; PBSC: Peripheral Blood Stem Cell

In a retrospective analysis by Kato et al. [50] patients who received salvage transplants from a different donor achieved engraftment, whereas the engraftment rate of HSCT from the same donor was 42.1 ± 11.8% (P = 0.02). Nevertheless, the estimated Overall Survival (OS) probability of the two groups did not reach statistical significance (P; 0.70).

Kongtim P et al. analyzed outcomes of patients with primary and secondary GF who received an unmanipulated haploidentical HSCT as salvage treatment and reported that using the same haploidentical donor was associated with poor OS with a hazard ratio (HR) of 2.90 (95% CI 1.07–7.92, P = 0.037) mainly due to increase in early nonrelapse mortality (NRM) [53].

In another retrospective study by Chewning et al. [51] the outcome of 16 consecutive patients who received a second HSCT following GF of initial HSCT was analyzed. Five of 10 patients transplanted from different donors survived compared with only 1 of 6 patients receiving stem cells from the same donor. Although the outcome was more favorable in the first group, this difference was not significant (P = 0.1).

Remarkably, for patients with suspected T-cell rejection as the cause of GF after haploidentical HSCT, using a different haploidentical donor with a different mismatched haplotype for the salvage transplant has been associated with a higher engraftment rate [51, 66].

### Second transplant from the same or different donor

A second transplant from a different donor or the same donor is still a matter of debate [27]. While changing to a different donor may contribute to successful engraftment, there are few studies about engraftment or survival outcomes of second HSCT based on donor choice with controversial results. In the setting of graft failure after HLA-matched HSCT, it is more common to use the same previous donor for a second transplant, because another well-matched donor is rarely available. However, the engraftment failure rate has been high when using the same donor [53]. Furthermore, the risk of re-collection from the initial donor in a short period of time should be considered [32].

### Autologous recovery

The recovery of host-hematopoiesis without evidence of disease relapse, known as autologous reconstitution (AR), is a rare event in patients with GF [59]. In a retrospective analysis of 1205 consecutive patients with severe aplastic anemia (SAA), conducted by the Aplastic Anemia Working Party of the European Group for Blood and Marrow Transplantation (EBMT–WPSAA), the cumulative incidence of AR was 4.2% (3.1–5.6) with an OS of 84% [76].

Rondon et al. [77] reported nine patients of 1726 allogeneic HSCT recipients who experienced autologous reconstitution after primary GF; seven following RIC regimens and two after myeloablative conditioning (MAC) regimen. Interestingly, patients with primary graft failure and AR had longer median survival compared to those who received a retransplant.

It is worth noting that all of our patients received a MAC regimen during their first allogeneic hematopoietic stem cell transplantation. However, two of them experienced autologous recovery.

In another retrospective cohort of 1,630 patients who underwent allogeneic HSCT for a malignant disease or severe AA, reported by Park et al. [59] primary and secondary GF occurred in 13 and 69 patients respectively. AR was observed in 11.6% (n = 8) of patients with an incidence of 0.49% of the overall study population and 11.6% among patients with secondary GF. The median time to onset of AR was 6.95 months (range, 2.3–16.7) after diagnosis of secondary GF. However, management with mobilized donor lymphocyte infusion (DLI) or rescue allogeneic HSCT was associated with a higher recovery rate compared to conservative care.

## Conclusion

Graft failure is one of the most important barriers to a successful transplant that can occur early after UCB, haploidentical, and HLA-mismatched donor transplants, as well as following nonmyeloablative or RIC regimens [40, 60, 62]. According to the clinician's clinical judgment, various therapeutic approaches may be considered after graft failure [61].

Although a second transplantation is almost the only salvage method, we illustrate that some pediatric patients with acute leukemia who experience graft failure after an allogeneic stem cell transplant using a MAC regimen may achieve long-term disease-free survival through autologous hematopoiesis recovery.

Considering the second transplant, it has been shown that non-myeloablative conditioning regimens for allogeneic hematopoietic cell transplantation have led to improved outcomes over the years, with reduced morbidity and mortality from infections, organ toxicity, and graft-versus-host disease [78].

Additionally, changing to a different donor has been identified as an important factor for successful engraftment in cases of graft failure, with a higher engraftment success rate observed when using a different donor [79]

Therefore, the use of a non-myeloablative conditioning regimen and a different donor for second transplants should be carefully considered based on the individual patient's condition and prospectively assessed for its potential benefits.

Overall, we acknowledge that further experiments are needed to strengthen the decision-making process in pediatric patients with acute leukemia who experience graft failure after HSCT.

## Data Availability

All supporting data and materials are included in the article and additional files.

## Abbreviations

GF: Graft failure
GvHD: Graft-versus-host disease
HSCT: Hematopoietic stem cell transplantation
MAC: Myeloablative conditioning
UCB: Umbilical cord blood
BM: Bone marrow
TCD: T-cell depleted
RIC: Reduced-intensity conditioning
CMV: Cytomegalovirus
HSCs: Hematopoietic stem cells
PB: Peripheral blood
CTL: Cytotoxic T lymphocytes
DSA: Donor-specific antibodies
PTCY: Post-transplant cyclophosphamide
NK: Natural killer
G-CSF: Granulocyte colony-stimulating factor
PBSC: Peripheral blood stem cells
WBC: White blood cell
STR: Short tandem repeat
STR-PCR: Short tandem repeat-polymerase chain reaction
DFS: Disease-free survival
TBI: Total body irradiation
AML: Acute myeloid leukemia
OS: Overall survival
HR: Hazard ratio
NRM: Nonrelapse mortality
Pre B Cell ALL: Precursor B cell acute lymphoblastic leukemia
HLA: Anti-human leukocyte antigen
MUD: Matched unrelated donors
MMUD: Mismatched unrelated donors
MRD: Measurable residual disease
MRD: Matched related donors
AR: Autologous reconstitution
SAA: Severe aplastic anemia
DLI: Donor lymphocyte infusion
